# Editorial: The DNA Replication Machinery as Therapeutic Targets

**DOI:** 10.3389/fmolb.2019.00035

**Published:** 2019-05-21

**Authors:** Andrew F. Gardner, Zvi Kelman

**Affiliations:** ^1^New England Biolabs, Inc., Ipswich, MA, United States; ^2^Biomolecular Labeling Laboratory, Institute for Bioscience and Biotechnology Research, Rockville, MD, United States; ^3^National Institute of Standards and Technology, Rockville, MD, United States

**Keywords:** DNA replication, replication stress, DNA polymerase, helicase, therapeutic targets

Chromosomal DNA replication is a process conserved through all domains of life. For accurate and efficient duplication of the genetic information, DNA replication components must work together in a highly regulated and coordinated fashion. Due to a central role in cell proliferation, the DNA replication machinery is an attractive therapeutic target for treating bacterial and viral infections, autoimmune disorders, and cancer. This eBook, entitled “The DNA Replication Machinery as Therapeutic Targets” explores how DNA replication factors serve as targets for new generations of therapies.

In all organisms, the chromosomal replication process starts at a specific chromosomal region called an origin of replication, at which origin-binding proteins (OBP) bind and locally unwind the duplex DNA. Additional proteins interact with the OBP-DNA complex and are responsible for the assembly of the DNA helicase around the DNA. Once assembled, the helicase unwinds the duplex in an ATP-dependent manner and forms the initial replication bubble. The exposed short regions of single-stranded DNA (ssDNA) at the replication bubble are coated with ssDNA-binding protein (SSB). DNA primase, DNA polymerases, and the rest of the replication machinery are recruited to the SSB-ssDNA complex to initiate bidirectional DNA synthesis. Due to the antiparallel nature of duplex DNA, and the unidirectionality of DNA polymerases, one strand of the chromosome is synthesized continuously (leading strand) while the other is copied discontinuously (lagging strand) as a series of Okazaki fragments (O'Donnell et al., 2013; Kelman and Kelman, 2014; Kunkel and Burgers, 2017). Although these processes are fundamentally conserved in the three domains: archaea, bacteria and eukarya; as well as viruses and bacteriophages, the proteins, and complexes involved differ (Makarova and Koonin, 2013).

Because the replication machinery is composed of a variety of core proteins and regulatory factors, disruption of any of the proteins involved will inhibit the replication process and/or its efficiency, and lead to replication stress. Replication stress is induced by endogenous factors such as dNTP depletion, DNA secondary structures or crosslinks, or by exogenous chemotherapies that damage DNA, such as cisplatin (Kitao et al., 2018). In human cells, replication stress occurs when DNA polymerases uncouples from the replisome and lags behind helicase unwinding (Zeman and Cimprich, 2014). As a result, long stretches of ssDNA are exposed at the replication fork. Replication protein A (RPA, the eukaryotic SSB) binds to the extended stretches of ssDNA, depleting free RPA in the cell and causing replication fork collapse (which may lead to DNA breakage and cell death). Therefore, inducing replication stress by disrupting the replication machinery or its regulation are an attractive strategies for drug design (O'Connor, 2015; Forment and O'Connor, 2018).

Each chapter in this eBook highlights a different therapeutic strategy to effectively target DNA replication. One approach to halt replication is to inhibit DNA polymerases via binding of small molecules to the active site. The three replicative DNA polymerases responsible for the duplication of chromosomal DNA in eukarya, DNA polymerases α, δ and ε (Pol α, Pol δ, and Pol ε), all belong to family B DNA polymerases. In order to develop nucleotide analogs as drugs, one needs to understand how DNA polymerases incorporate natural and modified nucleotides into DNA. The contribution by Daimon et al. adds to our understanding of the structure and function relationships of family B polymerases by solving the structure of two members of this family from *Aeropyrum pernix* (Daimon et al.). The incorporation of various classes of modified nucleotides and nucleotide terminators by another family B DNA polymerase from archaea is described by a review contributed by Gardner et al. These archaeal polymerases share sequence similarity with the eukaryotic enzymes and thus can serve as good model systems for the more complex eukaryotic replication (Makarova et al., 2014).

In addition, the use of nucleotide analogs to treat human autoimmune disorders and cancer is summarized in a contribution by Berdis. Young explores mitochondrial replication and how incorporation of certain nucleoside chain terminator inhibitors by Pol γ (the mitochondrial-specific polymerase) can lead to unintended toxicity by shutting down mitochondrial genome replication (Young). In addition, certain classes of compounds target Pol γ in cancer cells to inhibit mitochondrial replication with the potential to induce tumor cell death (Young).

Despite a central role in copying the chromosome, the inherent processivity of DNA polymerases is low, and only a few nucleotides are incorporated at a time. However, in the replication complex, high processivity DNA synthesis is conferred by a ring-shaped protein, referred to as the DNA polymerase sliding clamp, that encircles DNA and tethers the polymerase catalytic unit to the DNA for processive DNA synthesis (Indiani and O'Donnell, 2006). The sliding clamps cannot assemble themselves around the DNA and require an additional clamp loader complex that assembles the clamp around duplex DNA in an ATP-dependent manner. In addition to interacting with the polymerase, the sliding clamps of bacteria and eukarya also interact with dozens of other proteins involved in DNA replication, repair, and cell cycle progression (Vivona and Kelman, 2003). Therefore, inhibitors of the bacterial and eukaryal sliding clamps are being developed as anti-cancer and anti-bacterial drugs (Georgescu et al., 2008). The current knowledge on the development of sliding clamps inhibitors and their possible use as therapeutic agents is summarized in a review contribution by Altieri and Kelman.

Another key enzyme for cellular replication is the DNA helicase, the enzyme responsible for unwinding double-stranded DNA ahead of the replisome (Sakakibara et al., 2009). The contribution by Datta and Brosh describes the current state of the art in designing helicase inhibitors as anti-cancer drugs, and the issues surrounding the use of helicase inhibitors (Datta and Brosh). In eukarya, the replicative helicase is a complex of three components, the heterohexameric minichromosome maintenance (MCM), the tetrameric GINS complex and the Cdc45 protein. These form the CMG (Cdc45, MCM, GINS) complex (Onesti and MacNeill, 2013; O'Donnell and Li, 2018). Due to the essential role of CMG in chromosome replication, it is a prime target for anti-cancer drugs. The current efforts in the development of CMG inhibitors as anti-cancer drugs are summarized in a contribution by Seo and Kang. Instead of directly inhibiting an enzyme activity (such as DNA polymerase), another strategy is to deplete activity by downregulating gene expression or deregulating protein activity via the ubiquitination pathway (Jang et al.).

In addition, while some drugs are effective on their own, in other cases multiple replication factors can be targeted simultaneously to disrupt multiple pathways and lead to more efficient and effective treatment strategies. *Mycobacterium tuberculosis* is a pathogenic bacterium that is the etiological agent of tuberculosis (TB), which kills more than a million people a year (Bañuls et al., 2015). Reiche and coauthors summarize the current state of the development of drugs against the *M. tuberculosis* replication machinery, including drugs targeting the polymerase (Pol III), the sliding clamp, clamp loader, and other replication proteins (Reiche et al.). In addition, Reiche et al. demonstrate the increased effectiveness of a combination *M. tuberculosis* antibiotic strategy that depletes dNTP pools while inhibiting DNA polymerase activity (Reiche et al.). Another example of a combination strategy is the inhibition of DNA polymerase synthesis with nucleotide inhibitors in combination with DNA damaging agents to create DNA lesion that stall synthesis (Berdis).

## Remaining Challenges and Future Opportunities

Despite effective inhibitors of DNA replication proteins, eventual resistance to these inhibitors leads to tumor recurrence and remains a challenge for long-term therapeutic efficacy. Therefore, it will be important to continue to study molecular mechanisms of tumor resistance to DNA replication inhibitors. For example, DNA polymerase mutants that effectively remove nucleotide chain terminators can lead to drug resistance, as can upregulation of lesion bypass DNA polymerases (Berdis).

We anticipate that knowledge of DNA replication protein expression, regulation, and biochemical properties will continue to address these challenges and accelerate the development of novel strategies for effective treatment. To reach potential as therapeutic targets, more high-resolution structural information is needed for all replisome proteins and complexes to understand important replisome active site architectures and protein interactions. High resolution replisome structures will enable models for docking small molecules to inhibit enzyme activities and disrupt essential replisome interactions.

Finally, new molecular tools will accelerate identification of new DNA replication drugs targets. CRISPR-Cas9 genome engineering tools have revolutionized many scientific disciplines and offer a powerful method to alter genes by either modifying gene sequence or introducing insertions or deletions to knock out gene function (Doudna and Charpentier, 2014). Genome-wide CRISPR-Cas9 screens aim to disrupt all or a subset of genes in an organism to identify important genes in a pathway (Sánchez-Rivera and Jacks, 2015; Peters et al., 2016). CRISPR-Cas9 genome-wide screens can be adapted to identify novel factors that confer either resistance or sensitivity to DNA replication inhibitors. Knowledge of these factors may inform future therapeutic strategies to design new drug classes or enhance the efficacy of current therapies.

## Author Contributions

All authors listed have made a substantial, direct and intellectual contribution to the work, and approved it for publication.

### Conflict of Interest Statement

AG is employed and funded by New England Biolabs, Inc., a manufacturer and vendor of molecular biology reagents, including DNA replication and repair enzymes. This affiliation does not affect the author's impartiality, objectivity of data generation or its interpretation, adherence to journal standards and policies or availability of data. The remaining author declares that the research was conducted in the absence of any commercial or financial relationships that could be construed as a potential conflict of interest.
